# Availability of facility resources and services and infection-related maternal outcomes in the WHO Global Maternal Sepsis Study: a cross-sectional study

**DOI:** 10.1016/S2214-109X(21)00248-5

**Published:** 2021-07-21

**Authors:** Vanessa Brizuela, Cristina Cuesta, Gino Bartolelli, Abdulfetah Abdulkadir Abdosh, Sabina Abou Malham, Bouchra Assarag, Rigoberto Castro Banegas, Virginia Díaz, Faysal El-Kak, Mohamed El Sheikh, Aquilino M Pérez, João Paulo Souza, Mercedes Bonet, Edgardo Abalos, WHO GLOSS Research Group, Vanessa Brizuela, Cristina Cuesta, Gino Bartolelli, Abdulfetah Abdulkadir Abdosh, Sabina Abou Malham, Bouchra Assarag, Rigoberto Castro, Virginia Díaz, Faysal El Kak, Mohamed Elsheikh, Aquilino M. Pérez, João Paulo Souza, Mercedes Bonet, Edgardo Abalos, Mohammad Iqbal Aman, Bashir Noormal, Marisa Espinoza, Julia Pasquale, Charlotte Leroy, Kristien Roelens, Griet Vandenberghe, M. Christian Urlyss Agossou, Sourou Goufodji Keke, Christiane Tshabu Aguemon, Patricia Soledad Apaza Peralta, Víctor Conde Altamirano, Rosalinda Hernández Muñoz, José Guilherme Cecatti, Carolina Ribeiro do Valle, Vincent Batiene, Kadari Cisse, Henri Gautier Ouedraogo, Kannitha Cheang, Phirun Lam, Tung Rathavy, Elie Simo, Pierre-Marie Tebeu, Emah Irene Yakana, Javier Carvajal, María Fernanda Escobar, Paula Fernández, Lotte Berdiin Colmorn, Jens Langhoff-Roos, Wilson Mereci, Paola Vélez, Yasser Salah Eldin, Alaa Sultan, Alula M. Teklu, Dawit Worku, Richard Adanu, Philip Govule, Charles Noora Lwanga, William Enrique Arriaga Romero, María Guadalupe Flores Aceituno, Carolina Bustillo, Bredy Lara, Vijay Kumar, Vanita Suri, Sonia Trikha, Irene Cetin, Serena Donati, Carlo Personeni, Guldana Baimussanova, Saule Kabylova, Balgyn Sagyndykova, George Gwako, Alfred Osoti, Zahida Qureshi, Raisa Asylbasheva, Aigul Boobekova, Damira Seksenbaeva, Saad Eddine Itani, Meilė Minkauskienė, Diana Ramašauskaitė, Owen Chikhwaza, Luis Gadama, Eddie Malunga, Haoua Dembele, Hamadoun Sangho, Fanta Eliane Zerbo, Filiberto Dávila Serapio, Nazarea Herrera Maldonado, Juan I. Islas Castañeda, Tatiana Cauaus, Ala Curteanu, Victor Petrov, Yadamsuren Buyanjargal, Seded Khishgee, Bat-Erdene Lkhagvasuren, Amina Essolbi, Rachid Moulki, Zara Jaze, Arlete Mariano, Nafissa Bique Osman, Hla Mya Thway Einda, Thae Maung Maung, Khaing Nwe Tin, Tara Gurung, Amir Babu Shrestha, Sangeeta Shrestha, Kitty Bloemenkamp, Marcus J. Rijken, Thomas Van Den Akker, María Esther Estrada, Néstor J. Pavón Gómez, Olubukola Adesina, Chris Aimakhu, Bukola Fawole, Rizwana Chaudhri, Saima Hamid, M. Adnan Khan, María del Pilar Huatuco Hernández, Nelly M. Zavaleta Pimentel, Maria Lu Andal, Zenaida Dy Recidoro, Carolina Paula Martin, Mihaela Budianu, Lucian Puşcaşiu, Léopold Diouf, Dembo Guirassy, Philippe Marc Moreira, Miroslav Borovsky, Ladislav Kovac, Alexandra Kristufkova, Sylvia Cebekhulu, Laura Cornelissen, Priya Soma-Pillay, Vicenç Cararach, Marta López, María José Vidal Benedé, Hemali Jayakody, Kapila Jayaratne, Dhammica Rowel, Wisal Nabag, Sara Omer, Victoria Tsoy, Urunbish Uzakova, Dilrabo Yunusova, Thitiporn Siriwachirachai, Thumwadee Tangsiriwatthana, Catherine Dunlop, Marian Knight, David Lissauer, Jhon Roman, Gerardo Vitureira, Dinh Anh Tuan, Luong Ngoc Truong, Nghiem Thi Xuan Hanh, Mugove Madziyire, Thulani Magwali, Stephen Munjanja, Adama Baguiya, Mónica Chamillard, Bukola Fawole, Marian Knight, Seni Kouanda, Pisake Lumbiganon, Ashraf Nabhan, Ruta Nadisauskiene, Linda Bartlett, Fernando Bellissimo-Rodrigues, Shevin T. Jacob, Sadia Shakoor, Khalid Yunis, Liana Campodónico, Hugo Gamerro, Daniel Giordano, Fernando Althabe, A. Metin Gülmezoglu

**Affiliations:** a UNDP/UNFPA/UNICEF/WHO/World Bank Special Programme of Research, Development and Research Training in Human Reproduction (HRP), Department of Sexual and Reproductive Health and Research, WHO, Geneva, Switzerland; b School of Economics and Statistics, National University of Rosario, Rosario, Argentina; c St Paul's Hospital Millennium Medical College, Addis Ababa, Ethiopia; d Faculty of Medicine and Health Sciences, School of Nursing, Université de Sherbrooke, Longueuil, QC, Canada; e National School of Public Health, Rabat, Morocco; f Hospital Doctor Roberto Suazo Cordova, La Paz, Honduras; g Centro Rosarino de Estudios Perinatales (CREP), Rosario, Argentina; h Department of Obstetrics and Gynecology, American University of Beirut Medical Center, Beirut, Lebanon; i Faculty of Medicine, University of Khartoum, Khartoum, Sudan; j Centro Hospitalario Pereira Rossell, Montevideo, Uruguay; k Department of Social Medicine, Ribeirão Preto Medical School, Ribeirão Preto, Brazil

## Abstract

**Background:**

Infections are among the leading causes of maternal mortality and morbidity. The Global Maternal Sepsis and Neonatal Initiative, launched in 2016 by WHO and partners, sought to reduce the burden of maternal infections and sepsis and was the basis upon which the Global Maternal Sepsis Study (GLOSS) was implemented in 2017. In this Article, we aimed to describe the availability of facility resources and services and to analyse their association with maternal outcomes.

**Methods:**

GLOSS was a facility-based, prospective, 1-week inception cohort study implemented in 713 health-care facilities in 52 countries and included 2850 hospitalised pregnant or recently pregnant women with suspected or confirmed infections. All women admitted for or in hospital with suspected or confirmed infections during pregnancy, childbirth, post partum, or post abortion at any of the participating facilities between Nov 28 and Dec 4 were eligible for inclusion. In this study, we included all GLOSS participating facilities that collected facility-level data (446 of 713 facilities). We used data obtained from individual forms completed for each enrolled woman and their newborn babies by trained researchers who checked the medical records and from facility forms completed by hospital administrators for each participating facility. We described facilities according to country income level, compliance with providing core clinical interventions and services according to women's needs and reported availability, and severity of infection-related maternal outcomes. We used a logistic multilevel mixed model for assessing the association between facility characteristics and infection-related maternal outcomes.

**Findings:**

We included 446 facilities from 46 countries that enrolled 2560 women. We found a high availability of most services and resources needed for obstetric care and infection prevention. We found increased odds for severe maternal outcomes among women enrolled during the post-partum or post-abortion period from facilities located in low-income countries (adjusted odds ratio 1·84 [95% CI 1·05–3·22]) and among women enrolled during pregnancy or childbirth from non-urban facilities (adjusted odds ratio 2·44 [1·02–5·85]). Despite compliance being high overall, it was low with regards to measuring respiratory rate (85 [24%] of 355 facilities) and measuring pulse oximetry (184 [57%] of 325 facilities).

**Interpretation:**

While health-care facilities caring for pregnant and recently pregnant women with suspected or confirmed infections have access to a wide range of resources and interventions, worse maternal outcomes are seen among recently pregnant women located in low-income countries than among those in higher-income countries; this trend is similar for pregnant women. Compliance with cost-effective clinical practices and timely care of women with particular individual characteristics can potentially improve infection-related maternal outcomes.

**Funding:**

UNDP/UNFPA/UNICEF/WHO/World Bank Special Programme of Research, Development and Research Training in Human Reproduction, WHO, Merck for Mothers, and US Agency for International Development.

## Introduction

The past two decades have seen a great increase in institutional births across the world in an attempt to improve maternal and perinatal health outcomes.^1^, ^2^ However, maternal mortality and morbidity have not decreased as expected; every year, about 295 000 women die during and after pregnancy.^3^ Infections and sepsis continue to be among the leading causes of maternal mortality and morbidity, with the latest estimates for maternal sepsis accounting for 11% of all maternal deaths globally and contributing to thousands more each year.^4^

**Research in context**

**Evidence before this study**

We looked at data from two systematic reviews by WHO on maternal infections and did a literature search that included terms relating to maternal mortality and morbidity and facility characteristics, including water, sanitation, and hygiene, and infection prevention measures, with no language restrictions. Findings from the Global Maternal Sepsis Study (GLOSS), published in 2020, revealed that infections play a much larger role in global maternal mortality and morbidity than previously thought. In addition, GLOSS showed differences in maternal infection ratios depending on country income level, and revealed that about a third of women did not have a complete set of vital signs recorded at enrolment. Current guidance on the identification and early management of sepsis requires that particular clinical and laboratory assessments be made of women with suspected sepsis. A 2018 study looking at availability of facility resources in low-income and middle-income countries revealed the existing limitations for managing maternal sepsis.

**Added value of this study**

We found that individual women's characteristics known to be associated with poor maternal outcomes (eg, multiparity, pre-existing conditions, and pregnancies ending in abortion) are more probable predictors of severe maternal outcomes than facility characteristics. In addition, our study sheds light on low compliance with some essential interventions and clinical assessments that reveal shortcomings in the quality of care offered in health-care facilities. This is the first study to provide data on facility-based characteristics and compliance with particular assessments of clinical signs or laboratory tests from a global sample of health-care facilities enrolling women with suspected or confirmed maternal infections.

**Implications of all the available evidence**

Maternal infections have a larger impact on global maternal mortality and morbidity than previously thought. Certain characteristics of women are associated with worse maternal outcomes despite availability of resources and services at health-care facilities. Further research is needed to best understand why, when resources and services are seemingly available, women are still having poor outcomes, as well as how each individual facility resource and performance is linked to maternal outcomes. If health-care providers and decision makers are committed to reducing maternal mortality and morbidity, ensuring that resources are both available and used when needed remains crucial.

The Global Maternal Sepsis and Neonatal Initiative,^5^ launched in 2016 by WHO and partners, sought to reduce the burden of maternal infections and sepsis and was the basis upon which the Global Maternal Sepsis Study (GLOSS) was implemented in 2017.^6^, ^7^ GLOSS estimated the ratio of intrahospital maternal infections at 70 cases per 1000 livebirths and a ratio of 11 cases per 1000 livebirths for infection-related severe maternal outcomes (maternal death or near-miss).^7^ An important finding from GLOSS was that about a third of included women with suspected or confirmed infection did not have a complete set of vital signs recorded nor did they receive antimicrobials upon suspicion or confirmation of infection.

A key factor in maternal survival from infections or sepsis is a rapid response from the health system through early identification and management of the infection and its complications; integral to that is the availability of specific resources and services in the health-care facility necessary to make these assessments and treat the infection.^8^ Availability of essential services and resources for caring for women during pregnancy and the post-partum period, such as human resources; medical supplies and equipment; water, sanitation, and hygiene (WASH); and appropriate provision of care, is crucial.^9^, ^10^, ^11^

Understanding the association between facility characteristics and infection-related severe maternal outcomes can provide evidence to better inform facility-based guidance and policies aimed at improving maternal health. Building on findings on the frequency and management of maternal infections in health-care facilities, we sought to describe the services and resources available at GLOSS participating facilities and how these relate to infection-related severe maternal outcomes, adjusting for the individual characteristics of eligible women. We also looked at compliance with providing certain services and clinical interventions to assess whether women were receiving what they needed according to facilities' reported capacity.

## Methods

### Study design and participants

The protocol and initial findings from GLOSS were published elsewhere.^6^, ^7^ In short, GLOSS was a facility-based, prospective, 1-week inception cohort study implemented in 713 health-care facilities in 52 countries, including 2850 hospitalised pregnant or recently pregnant women with suspected or confirmed infections, accompanied by an awareness campaign.^12^

All women admitted for or in hospital with suspected or confirmed infections during pregnancy, childbirth, post partum, or post abortion at any of the participating facilities between Nov 28 and Dec 4, 2017, were eligible for inclusion in GLOSS. Written informed consent or a waiver of written consent (opt-out) was obtained depending on each country's requirements. Ethical approval for GLOSS was obtained from the WHO's Ethics Review Committee (protocol ID A65787), and from the ethics committees of the respective countries and facilities according to national regulations.

### Procedures

Data were collected using three different paper-based forms: individual forms for each enrolled woman and their newborn babies that were completed by trained researchers who checked the medical records; facility forms completed by hospital administrators for each participating facility; and area forms for each participating geographical area that were completed by study country coordinators. Individual-level data regarding identification and management of the infection were collected for up to 6 weeks or until discharge, transfer outside of study area, or death of the participant, as well as for their newborn babies up to 7 days after birth. These data included information relating to pregnancy status at enrolment to the study, clinical signs and symptoms during the first 3 days upon admission to hospital, and pregnancy and maternal outcomes. Facility-level data included information on location, administration, type of health-care institution (primary [level I], secondary [level II], or tertiary [level III]),^13^ and availability of specific services and interventions on the day that the form was completed, including clinical practices (eg, cultures, laboratory services, checking for clinical signs), obstetric care capacity, infection prevention measures, and availability of WASH, medicines, and treatments for women and neonates. Additionally, feasibility for detection of organ dysfunction, availability of protocols, and the presence of infection prevention and control committees were recorded for each facility. Data were entered manually into a web-based data management system developed for the study. Further details on the GLOSS protocol can be found in the appendix (pp 1–2). We present our data according to STROBE guidelines (appendix pp 3–5).

Of 713 facilities participating in GLOSS (maternity hospitals, referral or district hospitals, and general hospitals), we included those for which facility data were collected. We excluded facilities from countries not collecting these data (six countries, 267 facilities). We used data from the facility-level and individual-level forms for this analysis.

For the definition of compliance, we calculated the percentage of women within facilities who received interventions according to individual clinical need, by quartiles (≤25%, >25 to ≤50%, >50 to <75%, and ≥75%). Because we wanted to identify the facilities in the highest or lowest quartiles, we classified facilities as having low (up to 25% of women received a given intervention), intermediate (more than 25% but less than 75% of women received said interventions), or high compliance (at least 75% received said intervention). We defined country income level as low-income (LIC), lower-middle-income (LMIC), and upper-middle-income or high-income (UMHIC) using World Bank country classifications for 2018. To ensure standardisation throughout all the facilities with regards to capacity for basic emergency obstetric and newborn care, instead of relying on the form item that asked whether the facility had this capacity, we looked at facilities' reported availability of seven basic interventions: parenteral antibiotics, anticonvulsants, uterotonics, manual removal of placenta, removal of retained products, assisted vaginal delivery, and newborn resuscitation. Similarly, for comprehensive emergency obstetric and newborn care, we assessed for two additional interventions: surgery (ie, caesarean section) and blood transfusion. We created a caesarean index to identify the number of births delivered by caesarean section as a proportion of the total number of deliveries in 2016. For this index, we used a range of 13–17% as a reference, following guidance for suggested caesarean section rates.^14^

### Data analysis

We present proportions to report facility characteristics by country income level and by severity of maternal outcome, and compliance with measuring clinical signs or laboratory testing (ie, temperature, white blood cell count) as required according to suspicion or confirmation of infection.

We used a logistic multilevel mixed model using facility and individual characteristics to look at the association between these characteristics and infection-related maternal outcomes. We modelled infection-related maternal outcomes in two categories: severe maternal outcomes and non-severe maternal outcomes (ie, infections with complications and less severe infections). The reference category was non-severe maternal outcomes. Infection-related severe maternal outcome includes women with WHO near-miss criteria or maternal death. Infections with complications includes women who required an invasive procedure to treat the source of infection (eg, vacuum aspiration, dilatation and curettage, wound debridement, drainage [incision, percutaneous, culdotomy], laparotomy and lavage, other surgery), admission to intensive care unit or high dependency care, or transfer to another facility. All other women were considered to have less severe infections. We adjusted for key facility-level and individual-level variables in one stage on the basis of their clinical significance. For the list of variables included in the models, see the appendix (pp 1–2). We dichotomised compliance as high (≥75%) and not high (<75%) to allow for sufficient cases in each of the groups. To account for clustering and to control for a possible correlation between observations within each geographical area in participating countries, we included the country as a random effect in the models. We used two different models, one for women who were enrolled in the study during pregnancy or childbirth and another for women enrolled post partum or post abortion, given that the pathogenesis and clinical presentation of infections tend to be different between these two groups.^15^, ^16^ A consistency analysis was done to assess missing data; the analysis found that missing data were random and not systematic, so we included all observations in all further analyses. Statistical significance is reported at p<0·05. All statistical analyses were done using R, version 4.0.0 (R Foundation for Statistical Computing, Vienna, Austria).

### Role of the funding source

The funders of the study had no role in study design, data collection, data analysis, data interpretation, or writing of the report.

## Results

446 facilities from 46 GLOSS participating countries that enrolled 2560 women were included in the analyses; 145 (33%) were from UMHICs, 193 (43%) were from LMICs, and 108 (24%) were from LICs (table 1). Despite the overall high availability of certain practices and resources in all facilities, there were differences in some according to the income level of the country. Only 245 (58%) of 425 facilities with data available had an infectious disease specialist: 53 (50%) of 106 facilities in LICs and 72 (41%) of 176 facilities in LMICs compared with 120 (84%) of 143 in UMHICs. 299 (67%) of 444 facilities with data available reported availability of antibiotic or antimicrobial surveillance systems: 54 (50%) of 107 facilities in LICs, 128 (67%) of 192 facilities in LMICs, and 117 (81%) of 145 facilities in UMHICs. In UMHICs, 134 (92%) of 145 facilities were able to perform cultures compared with only 64 (59%) of 108 facilities in LICs. Although most facilities reported availability of sanitation and hygiene services (more than 70% of facilities for each country income level), waste management was reported as low in all facilities (247 [56%] of 443); 157 (35%) of 446 facilities reported no availability of incinerators, which are one of the components of waste management.

**Table 1:** Facility characteristics and availability of resources and services by country-level income in 46 GLOSS participating countries (n=446)

	**All (n=446)**	**Low-income (n=108)**	**Lower-middle-income (n=193)**	**Upper-middle or high-income (n=145)**
**Facility level**				
I	76/445 (17%)	21/108 (19%)	32/193 (17%)	23/144 (16%)
II	195/445 (44%)	36/108 (33%)	115/193 (60%)	44/144 (31%)
III	174/445 (39%)	51/108 (47%)	46/193 (24%)	77/144 (53%)
**Type of facility**				
Public	342/446 (77%)	81/108 (75%)	171/193 (89%)	90/145 (62%)
Non-public*	104/446 (23%)	27/108 (25%)	22/193 (11%)	55/145 (38%)
University hospital	201/434 (46%)	53/108 (49%)	66/184 (36%)	82/142 (58%)
**Location**				
Urban	353/445 (79%)	94/108 (87%)	121/192 (63%)	138/145 (95%)
Peri-urban or rural	92/445 (21%)	14/108 (13%)	71/192 (37%)	7/145 (5%)
**Facility size**				
Small (<1000 livebirths per year)	115/436 (26%)	11/108 (10%)	60/188 (32%)	44/140 (31%)
Medium (1000–2499 livebirths per year)	123/436 (28%)	33/108 (31%)	46/188 (24%)	44/140 (31%)
Medium to large (2500–4499 livebirths per year)	90/436 (21%)	20/108 (19%)	34/188 (18%)	36/140 (26%)
Large (≥4500 livebirths per year)	108/436 (25%)	44/108 (41%)	48/188 (26%)	16/140 (11%)
**Ability to perform certain clinical practices or tests**				
Perform cultures	330/446 (74%)	64/108 (59%)	132/193 (68%)	134/145 (92%)
Laboratory services	421/446 (94%)	100/108 (93%)	179/193 (93%)	142/145 (98%)
Checking for vital signs†	443/446 (99%)	108/108 (100%)	190/193 (98%)	145/145 (100%)
**Human resources availability**‡				
Midwife	389/442 (88%)	105/107 (98%)	169/190 (89%)	115/145 (79%)
Obstetrician	416/445 (93%)	103/108 (95%)	171/192 (89%)	142/145 (98%)
Other physician able to assist deliveries§	414/444 (93%)	93/108 (86%)	180/192 (94%)	141/144 (98%)
Anaesthesiologist	420/444 (95%)	101/107 (94%)	176/192 (92%)	143/145 (99%)
Infectious disease specialist	245/425 (58%)	53/106 (50%)	72/176 (41%)	120/143 (84%)
**Obstetric care capacity**				
BEmONC	366/444 (82%)	90/108 (83%)	155/193 (80%)	121/143 (85%)
CEmONC	353/444 (80%)	88/108 (82%)	144/193 (75%)	121/143 (85%)
Transfer services¶	341/446 (76%)	75/108 (69%)	152/193 (79%)	114/145 (79%)
**Infection prevention**				
Training programmes in infection prevention and control	360/443 (81%)	89/108 (82%)	155/192 (81%)	116/143 (81%)
Infection prevention and control committee	384/443 (87%)	83/107 (78%)	168/191 (88%)	133/145 (92%)
Antibiotic or antimicrobial surveillance	299/444 (67%)	54/107 (50%)	128/192 (67%)	117/145 (81%)
**WASH availability**				
Sanitation‖	355/442 (80%)	77/107 (72%)	144/191 (75%)	134/144 (93%)
Hygiene**	420/444 (95%)	97/107 (91%)	182/193 (94%)	141/144 (98%)
Waste management††	247/443 (56%)	67/107 (63%)	101/191 (53%)	79/145 (54%)

Data are n/N (%), where N is the number of facilities with data available. BEmONC=basic emergency obstetric and newborn care. CEmONC=comprehensive emergency obstetric and newborn care. WASH=water, sanitation, and hygiene.

* Includes private not-for-profit, non-governmental, faith-based, private for-profit, and other mixed-ownership organisations.

† Includes availability to measure body temperature and blood pressure.

‡ Includes professionals available at the facility 24 h a day, 7 days a week in the facility or on call (outside the facility), as well as professionals with partial availability.

§ Includes internal medicine specialists or any physician able to perform caesarean sections.

¶ Includes availability of a functional ambulance, petrol supplies for transport support, and a driver for patient transport.

‖ Facility reported availability of shower or washing facilities for women and usable improved toilet or latrine in the post-partum ward, and a sewerage system in the facility.

** Facility reported availability of water and soap for hand hygiene and hand sanitiser for staff in the place where childbirth is intended to take place.

†† Facility reported availability of puncture-proof boxes for sharps and three colour-coded and lined containers for infectious, non-infectious, and hazardous waste in the place where childbirth is intended to take place, and an incinerator in the facility.

The distribution of severity in maternal outcomes (less severe infections, infections with complications, and infection-related severe maternal outcomes) varied between countries with different income levels (table 2), with LICs having the highest proportion of women developing infection-related severe maternal outcomes (148 [20%] of 744). Women enrolled at level I facilities were more likely to have less severe infections (155 [76%] of 204) than were those enrolled at level II (545 [67%] of 812) or level III facilities (877 [57%] of 1540). The proportion of women with severe maternal outcomes was slightly higher in medium-to-large facilities (96 [16%] 588; *vs* other facility sizes), public facilities (346 [15%] of 2289; *vs* non-public), teaching facilities (294 [16%] of 1835; *vs* non-teaching facilities), and level III facilities (269 [17%] of 1540; *vs* other levels). Overall, more than 50% of the sample had less severe infections. The distribution of severity in maternal outcomes according to availability of certain clinical practices or resources (table 3) followed a similar pattern across all domains.

**Table 2:** Maternal outcomes according to country income classification and facility characteristics (n=2560)

	**All women**	**Less severe infections**	**Infections with complications**	**Infection-related SMO**
**Country income level**				
Low income	744	379 (51%)	217 (29%)	148 (20%)
Low-middle income	1050	677 (64%)	229 (22%)	144 (14%)
Upper-middle and high income	766	524 (68%)	153 (20%)	89 (12%)
**Facility level**				
I	204	155 (76%)	26 (13%)	23 (11%)
II	812	545 (67%)	178 (22%)	89 (11%)
III	1540	877 (57%)	394 (26%)	269 (17%)
**Type of facility**				
Public	2289	1405 (61%)	538 (24%)	346 (15%)
Non-public*	271	175 (65%)	61 (23%)	35 (13%)
**Teaching facility**				
Yes	1835	1098 (60%)	443 (24%)	294 (16%)
No	657	437 (67%)	140 (21%)	80 (12%)
**Location**				
Urban	2331	1431 (61%)	552 (24%)	348 (15%)
Peri-urban or rural	226	147 (65%)	46 (20%)	33 (15%)
**Facility size**				
Small (<1000 livebirths per year)	184	122 (66%)	39 (21%)	23 (13%)
Medium (1000–2499 livebirths per year)	318	213 (67%)	63 (20%)	42 (13%)
Medium to large (2500–4499 livebirths per year)	588	355 (60%)	137 (23%)	96 (16%)
Large (≥4500 livebirths per year)	1412	846 (60%)	352 (25%)	214 (15%)

Data are n (%). Country income classifications are as per World Bank classification in 2018. SMO=severe maternal outcome (includes maternal near-miss and maternal death).

* Includes private not-for-profit, non-governmental, faith-based, private for-profit, and other mixed-ownership organisations.

**Table 3:** Maternal outcome according to availability of clinical practices, obstetric care capacity, infection prevention, and WASH measures as reported by the facility (n=2560)

		**All women**	**Less severe infections**	**Infections with complications**	**Infection-related SMO**
**Ability to do certain clinical practices or tests**					
Cultures					
	Yes	2114	1304 (62%)	495 (23%)	315 (15%)
	No	446	276 (62%)	104 (23%)	66 (15%)
Laboratory services					
	Yes	2506	1543 (62%)	584 (23%)	379 (15%)
	No	54	37 (69%)	15 (28%)	2 (4%)
**Obstetric care capacity**					
BEmONC					
	Yes	2280	1388 (61%)	551 (24%)	341 (15%)
	No	253	170 (67%)	46 (18%)	37 (15%)
CEmONC					
	Yes	2253	1371 (61%)	545 (24%)	337 (15%)
	No	280	187 (67%)	52 (19%)	41 (15%)
Transfer services*					
	Yes	2130	1316 (62%)	486 (23%)	328 (15%)
	No	430	264 (61%)	113 (26%)	53 (12%)
**Infection prevention**					
Training programmes in infection prevention and control					
	Yes	2233	1354 (61%)	538 (24%)	341 (15%)
	No	299	203 (68%)	59 (20%)	37 (12%)
Infection prevention and control committee					
	Yes	2341	1440 (62%)	554 (24%)	347 (15%)
	No	209	132 (63%)	43 (21%)	34 (16%)
Antibiotic or antimicrobial surveillance					
	Yes	1783	1087 (61%)	420 (24%)	276 (15%)
	No	773	492 (64%)	177 (23%)	104 (13%)
**WASH availability**					
Sanitation†					
	Yes	2179	1351 (62%)	502 (23%)	326 (15%)
	No	356	220 (62%)	85 (24%)	51 (14%)
Hygiene‡					
	Yes	2365	1474 (62%)	539 (23%)	352 (15%)
	No	181	105 (58%)	49 (27%)	27 (15%)
Waste management§					
	Yes	1427	887 (62%)	328 (23%)	212 (15%)
	No	1098	683 (62%)	255 (23%)	160 (15%)

Data are n (%). BEmONC=basic emergency obstetric and newborn care. CEmONC=comprehensive emergency obstetric and newborn care. SMO=severe maternal outcome (includes maternal near-miss and death). WASH=water, sanitation, and hygiene.

* Includes availability of a functional ambulance, petrol supplies for transport support, and a driver for patient transport.

† Facility reported availability of shower or washing facilities for women and usable improved toilet or latrine in the post-partum ward, and a sewerage system in the facility.

‡ Facility reported availability of water and soap for hand hygiene and hand sanitiser for staff in the place where childbirth is intended to take place.

§ Facility reported availability of puncture-proof boxes for sharps and three colour-coded and lined containers for infectious, non-infectious, and hazardous waste in the place where childbirth is intended to take place, and an incinerator in the facility.

**Figure 1:**
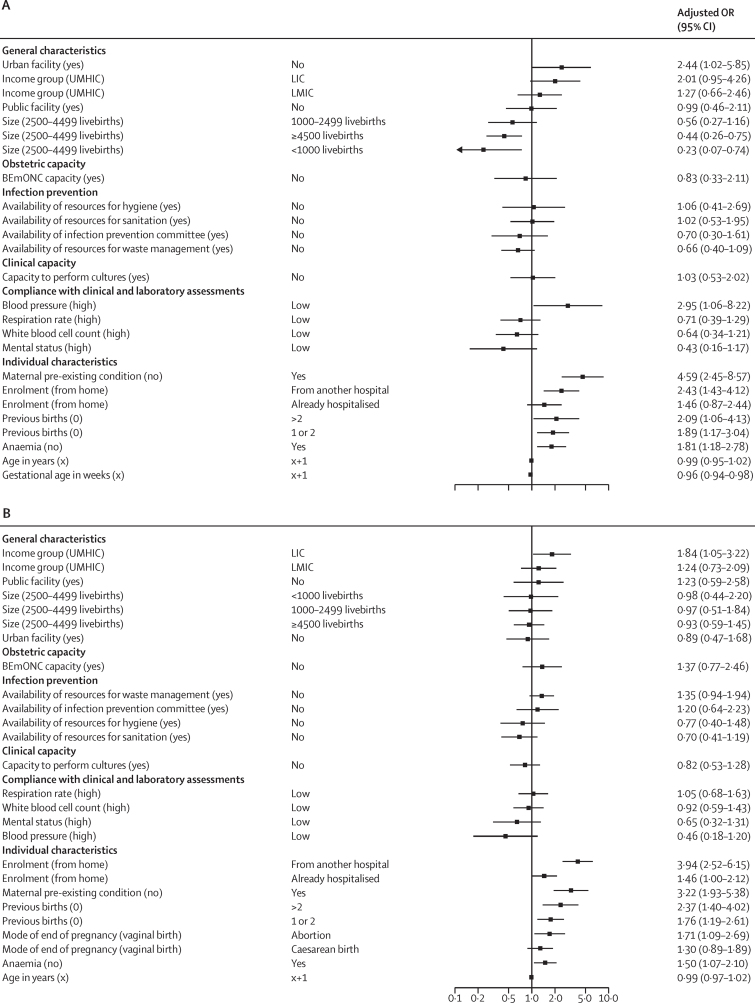
Association between facility and individual characteristics and infection-related severe maternal outcomes (A) Women enrolled during pregnancy or childbirth (n=1100). (B) Women enrolled during the post-partum or post-abortion period (n=1252). Characteristics are presented with the reference variable in parentheses. LIC=low-income country. LMIC=lower-middle-income country. UMHIC=upper-middle or high-income country. BEmONC=basic emergency obstetric and neonatal care. OR=odds ratio.

Our analysis of the association between facility and individual characteristics with infection-related severe maternal outcomes showed that individual women's characteristics were more likely to be associated with infection-related severe maternal outcomes than facility characteristics or compliance with clinical and laboratory assessments (figure 1). The odds of having an infection-related severe maternal outcome were higher among all women if they had a pre-existing condition, anaemia, or previous births than if they did not, or if they were referred from another facility. Women whose pregnancies ended in abortion had increased odds of having a severe maternal outcome compared with women whose pregnancies ended in a vaginal birth (adjusted odds ratio [OR] 1·71 [95% CI 1·09–2·69]). The only facility characteristic that was associated with a severe maternal outcome among women in the post-partum or post-abortion period was whether the facility was located in a LIC versus a UMHIC (1·84 [1·05–3·22]). Pregnant women or women in labour enrolled in non-urban facilities had increased odds of having a severe maternal outcome (2·44 [1·02–5·85]), whereas those enrolled in very small (0·23 [0·07–0·74]) or very large facilities (0·44 [0·26–0·75]) had decreased odds of severe maternal outcomes. For the full results, including crude and adjusted ORs, see the appendix (pp 6–9).

**Figure 2:**
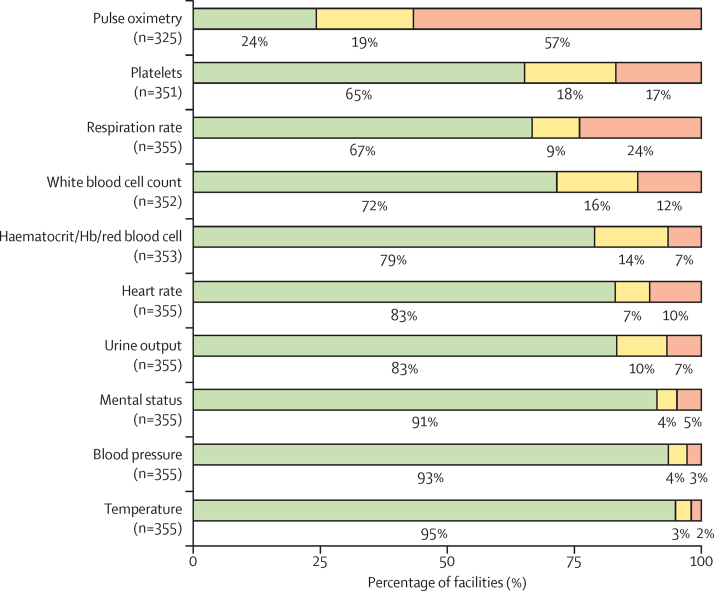
Facility compliance with clinical and laboratory assessments at study eligibility Compliance is reported as the percentage of facilities measuring clinical signs or doing laboratory tests in eligible women, ordered by increasing frequency of high compliance. High compliance (green) corresponds to facilities in which at least 75% of women got their vital signs checked; intermediate compliance (yellow) corresponds to facilities in which more than 25% and less than 75% of women got their vital signs checked; low compliance (red) corresponds to facilities in which up to 25% of women got their vital signs checked (n is the number of facilities where the service is offered and was supplied to women at study eligibility). Hb=haemoglobin.

Lastly, our analysis of compliance with particular clinical interventions and assessments showed that this was mostly high for all, except for compliance with measuring women's respiratory rate, which was low in 85 (24%) of 355 facilities, and compliance with pulse oximetry, which was low in 184 (57%) of 325 facilities (figure 2).

## Discussion

To the best of our knowledge, this is the first study to look at characteristics of more than 400 facilities located in 46 countries with varying income levels and the association of these characteristics with infection-related maternal outcomes. Most GLOSS participating facilities reported an ability to provide core clinical services and an availability of resources necessary to identify and manage maternal infections. Individual women's characteristics were more likely to be associated with severe maternal outcomes than facility characteristics, except for country income level and only in a subset of the sample. Compliance with particular interventions and assessments was mostly high, except for compliance with measuring respiratory rate and pulse oximetry when clinically required.

Similar to what others have shown, we found that women hospitalised in facilities located in LICs fared worse than those in higher-income countries.^4^, ^17^ Even though the result was not significant for pregnant women, this was probably due to insufficient power rather than an absence of association. The difference regarding the association between facility size and location and maternal outcomes, according to whether women were pregnant or recently pregnant, might be a reflection of the different facilities in which these two groups of women would be typically cared for. However, overall associations between facility characteristics and severe maternal outcomes would not say much about whether the women needing the services and resources received them. It is probable that the facilities caring for hospitalised women with suspected or confirmed infections are those most equipped with the necessary resources to address infections. This finding, in relation to those from a previous multicountry study that showed that the availability of essential interventions did not necessarily result in improved maternal outcomes, calls for cautious interpretation.^10^

We found that individual characteristics are more clearly associated with infection-related severe maternal outcomes than facility characteristics. Although this is an expected outcome, especially because we only considered individual characteristics that the literature and our previous analysis for GLOSS have shown are associated with maternal mortality or near-miss,^4^, ^7^, ^10^ it calls for health-care providers to ensure pregnant or recently pregnant women with suspected or confirmed infections are identified and managed promptly. Relatedly, results from the evaluation of the GLOSS awareness campaign implemented in all participating facilities revealed that health-care providers' baseline levels of certain knowledge and confidence with knowing how to care for maternal sepsis were low.^18^ In addition, the strong link we found between anaemia and severe maternal outcomes reinforces the importance of good antenatal care.^19^ We found that the association between caesarean section delivery and infection-related severe maternal outcomes was not significant, which might either be an absence of an association or a reflection of the sample size. In fact, there is evidence indicating that caesarean sections without medical indication might increase the risk of maternal morbidity and mortality in some settings.^20^, ^21^ There is clearly still work to be done with regards to prevention measures and appropriate and timely management of infections, particularly in women with underlying conditions or whose pregnancies end in abortion.

Our analysis of compliance sought to answer whether the availability of resources and services resulted in receipt of those services by women in need. Despite seemingly high availability of resources and clinical practices, compliance revealed limitations with regards to the timely identification and prompt management of women with suspected or confirmed infection. The fact that some essential, low-cost assessments, such as checking for respiratory rate, heart rate, or urine output, are not being done to all hospitalised women with suspected or confirmed infections in all GLOSS facilities is alarming. This is especially so given that the facilities participating in GLOSS were selected because they could admit women with infection and because existing warning signals and scoring systems for sepsis require that these essential measures be taken (eg, the quick sequential organ failure assessment [qSOFA], modified early obstetric warning system [MEOWS], or UK Sepsis Trust tools).^22^, ^23^, ^24^ Evidence has shown that even when the resources are at facilities' disposal, such as pulse oximetry for measuring oxygen saturation in low resource settings, these are not always readily available to all patients needing them or they are not functional.^25^

Our study provides real-life evidence of the link between availability of resources and services necessary to prevent, identify, and manage maternal infections in health-care facilities and infection-related severe maternal outcomes. However, this study has some limitations. First, data on facilities were obtained from facility forms, which included information reported by facility administrators on the day of form completion; we did not collect observational data nor data at more than one timepoint. Relatedly, the quality and veracity of the data included in the medical records was not validated or checked, meaning some of our results could be biased. Second, we measured facility compliance as a percentage of women who received an intervention according to individual need and facility availability, which is one way in which to assess whether women are receiving the care they require when infection is suspected or confirmed, but we cannot extend this assumption for women hospitalised for other conditions. Third, for the construction of the compliance indicator, we took the best measurement of three possible measures collected for assessment of particular clinical signs and laboratory testing, meaning our estimations might be better than real-life compliance. Fourth, we present data on the availability of health-care providers able to assist in a delivery but not whether these providers were sufficient for the number of women they cared for nor the specificity of each provider's expertise and the required need per woman. And lastly, the fact that we were unable to include data from 267 facilities in high-income countries might limit the generalisability of our findings; however, our analysis included 144 facilities from UMHICs.

Our analysis calls for further research to best understand why, when resources and services are seemingly available, women are still having poor outcomes, as well as how each individual facility's resources and performance are linked to maternal outcomes. Ensuring that resources are both available and used when needed, as well as that staff are trained to swiftly identify women at risk for severe maternal infections and follow clinical guidelines or use specific care bundles^26^ for the management of women with suspected or confirmed infections remains crucial. This approach might also require training on the importance of both assessing for vital signs and recording these measures in patient clinical records. Our study provides evidence relevant to clinicians, hospital managers, and policy makers that particular characteristics of women are associated with worse maternal outcomes despite the availability of resources and services. Although these findings are specific to GLOSS facilities, they are probably relevant to all facilities that admit pregnant or recently pregnant women with a suspected or confirmed infection. If health-care providers and decision makers are committed to reducing maternal mortality and morbidity, it is essential that they act on this evidence. Our findings, together with data on the frequency and management of individual infections^7^ and the factors that influence provider identification and management of maternal infections,^12^ speak to the need for ensuring facility personnel are using the available resources with the women they care for.

Although health-care facilities caring for pregnant and recently pregnant women with suspected or confirmed infections have access to a wide range of human resources and interventions, worse maternal outcomes are seen among recently pregnant women located in LICs than among those in higher-income countries, and this is possibly also true for pregnant women. Compliance with cost-effective clinical practices and interventions and timely care of women with particular individual characteristics can potentially improve maternal outcomes related to infections.

## Data sharing

The data used for this analysis can be made available upon reasonable request, in accordance with the GLOSS Research Group data sharing policy and WHO policy of data use and data sharing. For further information, contact the corresponding author.

## Declaration of interests

We declare no competing interests.
