# Supplementary material for: Availability of facility resources and services and infection-related maternal outcomes in the WHO Global Maternal Sepsis Study: a cross-sectional study
Source: Lancet Glob Health. 2021 Jul 21;9(9):e1252–61. doi: 10.1016/S2214-109X(21)00248-5 (PMC8370881; doi:10.1016/S2214-109X(21)00248-5)
Supplement: Supplementary appendix [file mmc1.pdf]

THE LANCET
Global Health

Supplementary appendix

This appendix formed part of the original submission and has been peer reviewed. We post it as supplied by the authors.

Supplement to: Brizuela V, Cuesta C, Bartolelli, G, et al. Availability of facility resources and services and infection-related maternal outcomes in the WHO Global Maternal Sepsis Study: a cross-sectional study. *Lancet Glob Health* 2021; published online July 14. http://dx.doi.org/10.1016/S2214-109X(21)00248-5.

APPENDIX

1. Key methods and variables used for GLOSS and current analysis

**GLOSS inclusion criteria**<br>a- Eligible geographical areas within each country	• Known number of inhabitants and of at least two million<br>• At least 30% institutional birth coverage<br>• At least 15,000 births/year total among all healthcare facilities within the area<br>• At least one referral health facility able to provide comprehensive emergency obstetric and neonatal care
b- Eligible facilities	• Provides obstetric, midwifery or post-abortion care (i.e. admits women for birth (live birth or stillbirth) or abortion (spontaneous or induced)/post-abortion care)<br>• Has an emergency room, adult ward, intensive care unit, or special care unit or any other setting where women can be admitted due to complications during pregnancy, childbirth or during the first 42 days after the end of pregnancy<br><br>For maternity hospitals:<br>o Minimal number of 1,000 births per facility/per year OR minimal level of care (e.g. tertiary and secondary level, national and district hospitals)<br>o About 80% coverage of all facility-based births in the geographical area
c- Eligible women	Pregnant or recently pregnant women hospitalized in any of the participating facilities with any of the following:<br>• Any suspected or confirmed infection during the current hospital stay (primary admission or readmission)<br>• Any clinical signs suggestive of infection (e.g. fever)<br>• Request for any body fluid culture or swab specimens<br>• Non-prophylactic use of antibiotics or other antimicrobial drugs at admission or during hospital stay<br>• Any health care-associated infections<br>• Any unexplained organ-dysfunction<br>• Any maternal death
**Inclusion criteria for this analysis**	• GLOSS participating facilities that collected facility-level data
**Types of analyses conducted**	• Proportions for facility characteristics by country income and severity of maternal outcome<br>• Compliance with clinical and laboratory assessments as low, intermediate, or high<br>• Logistic multilevel mixed model for the association between facility and women characteristics and severe maternal outcome
**Variables included in the models**	**Facility level variables**<br>Categories in between brackets. If none stated, then assume yes/no.<br>*General characteristics*<br>• Country income group (low-income; middle-income; upper-middle/high-income)

- Facility type (public; not public)
- Location (urban; peri-urban/rural)
- Size in live births/year (<1,000, ≥ 1000 & ≤ 2499, ≥ 2500 & ≤ 4499, ≥ 4500)

Obstetric capacity

- BEmONC

Infection prevention

- Availability of infection prevention committee
- Availability of resources for sanitation
- Availability of resources for hygiene
- Availability of resources for waste management

Clinical capacity

- Capacity to perform cultures

Compliance with clinical and laboratory assessments

- Compliance with clinical and laboratory assessments at enrolment in the study (low; high)

Individual level variables

Categories in between brackets. If none stated, then assume yes/no.

- Age (continuous)
- Gestational age (continuous)
- Pre-existing conditions
- Anaemia
- Previous births (0; 1 or 2; >2)
- Provenance of woman at enrolment (home; already hospitalized; transferred from another hospital)
- Mode of end of pregnancy (abortion; vaginal birth; caesarean birth)

Source: Bonet M, Souza JP, Abalos E, Fawole B, Knight M, Kouanda S, Lumbiganon P, Nabhan A, Nadisauskiene R, Brizuela V, Gülmezoglu AM. The global maternal sepsis study and awareness campaign (GLOSS): study protocol. Reproductive health. 2018 Dec;15(1):1-7.

2. STROBE checklist used for this analysis

	Item	Recommendation	Page	Relevant section in manuscript
**Title and abstract**	1	(*a*) Indicate the study's design with a commonly used term in the title or the abstract	Title page	Title: "Availability of facility resources and services and infection-related maternal outcomes in the WHO Global Maternal Sepsis Study: a cross-sectional study"
		(*b*) Provide in the abstract an informative and balanced summary of what was done and what was found	3-4	Abstract included at the beginning summarizing the background, methods, findings, and interpretation.
**Introduction**				
Background/rationale	2	Explain the scientific background and rationale for the investigation being reported	5-6	Introduction section
Objectives	3	State specific objectives, including any prespecified hypotheses	6	Last paragraph in introduction section
**Methods**				
Study design	4	Present key elements of study design early in the paper	6-8	Methods section
Setting	5	Describe the setting, locations, and relevant dates, including periods of recruitment, exposure, follow-up, and data collection	6-8	Methods section and appendix 1
Participants	6	(*a*) Give the eligibility criteria, and the sources and methods of selection of participants	6-8	Methods section and appendix 1
Variables	7	Clearly define all outcomes, exposures, predictors, potential confounders, and effect modifiers. Give diagnostic criteria, if applicable	8-9	Data analysis sub-section and appendix 1
Data sources/ measurement	8*	For each variable of interest, give sources of data and details of methods of assessment (measurement). Describe comparability of assessment methods if there is more than one group	6-9	Methods section and appendix 1
Bias	9	Describe any efforts to address potential sources of bias	N/A	
Study size	10	Explain how the study size was arrived at	6-8	Methods section and appendix 1
Quantitative variables	11	Explain how quantitative variables were handled in the analyses. If applicable, describe which groupings were chosen and why	8-9	Data analysis sub-section
Statistical methods	12	(*a*) Describe all statistical methods, including those used to control for confounding	8-9	Data analysis sub-section

		(*b*) Describe any methods used to examine subgroups and interactions	N/A	
		(*c*) Explain how missing data were addressed	9	Data analysis sub-section
		(*d*) If applicable, describe analytical methods taking account of sampling strategy	N/A	
		(*e*) Describe any sensitivity analyses	N/A	
**Results**				
Participants	13*	(a) Report numbers of individuals at each stage of study—eg numbers potentially eligible, examined for eligibility, confirmed eligible, included in the study, completing follow-up, and analysed	9-11	Results section and appendix 1
		(b) Give reasons for non-participation at each stage	N/A	
		(c) Consider use of a flow diagram	N/A	
Descriptive data	14*	(a) Give characteristics of study participants (eg demographic, clinical, social) and information on exposures and potential confounders	10-11	Table 1
		(b) Indicate number of participants with missing data for each variable of interest	10-11	Table 1
Outcome data	15*	Report numbers of outcome events or summary measures	12-14	Tables 2a and 2b
Main results	16	(*a*) Give unadjusted estimates and, if applicable, confounder-adjusted estimates and their precision (eg, 95% confidence interval). Make clear which confounders were adjusted for and why they were included	15-16	Results sub-section presenting adjusted odds ratios, Figure 1, and appendices 3a and 3b for adjusted and unadjusted odds ratios
		(*b*) Report category boundaries when continuous variables were categorized	N/A	
		(*c*) If relevant, consider translating estimates of relative risk into absolute risk for a meaningful time period	N/A	
Other analyses	17	Report other analyses done—eg analyses of subgroups and interactions, and sensitivity analyses	N/A	
**Discussion**				
Key results	18	Summarise key results with reference to study objectives	15	First paragraph of Discussion section
Limitations	19	Discuss limitations of the study, taking into account sources of potential bias or imprecision. Discuss both direction and magnitude of any potential bias	17	Limitations of this study

Interpretation	20	Give a cautious overall interpretation of results considering objectives, limitations, multiplicity of analyses, results from similar studies, and other relevant evidence	15-18	Discussion section relevant sections and interaction with the literature
Generalisability	21	Discuss the generalisability (external validity) of the study results	18	Second to last paragraph of discussion section
**Other information**				
Funding	22	Give the source of funding and the role of the funders for the present study and, if applicable, for the original study on which the present article is based	4, 9 & 21-22	Abstract, end of Methods section, and Acknowledgements section

3a. Association between facility and individual characteristics and infection-related severe maternal outcomes (SMO)* among women enrolled during pregnancy or childbirth, presented as proportions and as crude and adjusted odds ratios with 95% confidence intervals (N = 1,100)

Variable		SMO/women	aOR [95% CI]	cOR [95% CI]
**General characteristics**				
Urban facility	No	11/75	2.44 [1.02 - 5.85]	1.32 [0.65 - 2.68]
	Yes	111/1025	ref	ref
Income group	LIC	37/244	2.01 [0.95 - 4.26]	1.61 [0.89 - 2.89]
	LMIC	45/452	1.27 [0.66 - 2.46]	1.01 [0.58 - 1.75]
	UMHIC	40/404	ref	ref
Public facility	No	19/162	0.99 [0.46 - 2.11]	0.76 [0.39 - 1.49]
	Yes	103/938	ref	ref
Size	1000 - 2499 livebirths	15/154	0.56 [0.27 - 1.16]	0.51 [0.27 - 0.98]
	≥ 4500 livebirths	57/568	0.44 [0.26 - 0.75]	0.59 [0.38 - 0.93]
	< 1000 livebirths	5/83	0.23 [0.07 - 0.74]	0.33 [0.12 - 0.89]
	2500 - 4499 livebirths	45/295	ref	ref
**Obstetric capacity**				
BEmONC capacity	No	7/110	0.83 [0.33 - 2.11]	0.50 [0.22 - 1.13]
	Yes	115/990	ref	ref
**Infection prevention**				
Availability of resources for hygiene	No	8/68	1.06 [0.41 - 2.69]	0.98 [0.43 - 2.23]
	Yes	114/1032	ref	ref
Availability of resources for sanitation	No	17/141	1.02 [0.53 - 1.95]	1.07 [0.60 - 1.90]
	Yes	105/959	ref	ref
Availability of infection prevention committee	No	11/87	0.70 [0.30 - 1.61]	1.09 [0.54 - 2.22]
	Yes	111/1013	ref	ref
Availability of resources for waste management	No	41/453	0.66 [0.40 - 1.09]	0.66 [0.43 - 1.01]
	Yes	81/647	ref	ref
**Clinical capacity**				
Capacity to perform cultures	No	18/162	1.03 [0.53 - 2.02]	0.95 [0.53 - 1.68]
	Yes	104/938	ref	ref
**Compliance with clinical and laboratory assessments**				
Blood pressure[1]	Low	9/66	2.95 [1.06 - 8.22]	1.28 [0.56 - 2.92]
	High	113/1034	ref	ref
Respiration rate[1]	Low	29/278	0.71 [0.39 - 1.29]	0.87 [0.53 - 1.44]
	High	93/822	ref	ref
White blood cell count	Low	21/225	0.64 [0.34 - 1.21]	0.61 [0.35 - 1.07]
	High	101/875	ref	ref
Mental status[1]	Low	6/84	0.43 [0.16 - 1.17]	0.53 [0.21 - 1.31]
	High	116/1016	ref	ref
**Individual characteristics**				
Maternal pre-existing condition	No	100/1029	ref	ref
	Yes	22/71	4.59 [2.45 - 8.57]	4.15 [2.37 - 7.26]

Enrolment	From another hospital	33/181	2.43 [1.43 - 4.12]	2.25 [1.40 - 3.64]
	Already hospitalized	28/251	1.46 [0.87 - 2.44]	1.28 [0.79 - 2.06]
	From home	61/668	ref	ref
Previous births	> 2	22/162	2.09 [1.06 - 4.13]	1.80 [1.02 - 3.15]
	1 or 2	59/434	1.89 [1.17 - 3.04]	1.79 [1.17 - 2.73]
	0	41/504	ref	ref
Anaemia	No	68/756	ref	ref
	Yes	54/344	1.81 [1.18 - 2.78]	1.97 [1.32 - 2.94]
Age in years (x)[2]	x+1	-	0.99 [0.95 - 1.02]	1.01 [0.98 - 1.04]
Gestational age in weeks (x)[3]	x+1	-	0.96 [0.94 - 0.98]	0.97 [0.95 - 0.99]

*Severe maternal outcome includes maternal near-miss and death

cOR: crude odds ratio
aOR: adjusted odds ratio
CI: confidence interval
LIC: low-income country; LMIC: lower-middle income country; UMHIC: upper-middle/high-income country
BEmONC: basic emergency obstetric and newborn care

[1] Measures used for the qSOFA score for identifying high-risk patients for sepsis-related mortality
[2] As compared to women one year younger
[3] As compared to women with a gestational age of one week less

3b. Association between facility and individual characteristics and infection-related severe maternal outcomes (SMO)* among women enrolled during postpartum or post-abortion period, presented as proportions and as crude and adjusted odds ratios with 95% confidence intervals (N = 1,252)

**Variable**		**SMO/women**	**aOR [95% CI]**	**cOR [95% CI]**
**General characteristics**				
Income group	LIC	100/443	1.84 [1.05 - 3.22]	1.77 [1.03 - 3.06]
	LMIC	79/506	1.24 [0.73 - 2.09]	1.11 [0.64 - 1.92]
	UMHIC	42/303	ref	ref
Public facility	No	14/83	1.23 [0.59 - 2.58]	0.96 [0.51 - 1.82]
	Yes	207/1169	ref	ref
Size	< 1000 livebirths	14/78	0.98 [0.44 - 2.20]	1.08 [0.52 - 2.22]
	1000 - 2499 livebirths	22/129	0.97 [0.51 - 1.84]	1.12 [0.61 - 2.05]
	2500 - 4499 livebirths	45/275	ref	ref
	≥ 4500 livebirths	140/770	0.93 [0.59 - 1.45]	1.06 [0.71 - 1.60]
Urban facility	No	17/124	0.89 [0.47 - 1.68]	0.64 [0.35 - 1.15]
	Yes	204/1128	ref	ref
Obstetric capacity				
BEmONC capacity	No	24/111	1.37 [0.77 - 2.46]	1.27 [0.75 - 2.15]
	Yes	197/1141	ref	ref
Infection prevention				
Availability of resources for waste management	No	105/543	1.35 [0.94 - 1.94]	1.17 [0.84 - 1.63]
	Yes	116/709	ref	ref
Availability of infection prevention committee	No	19/101	1.20 [0.64 - 2.23]	1.07 [0.60 - 1.91]
	Yes	202/1151	ref	ref
Availability of resources for hygiene	No	17/107	0.77 [0.40 - 1.48]	0.71 [0.37 - 1.34]
	Yes	204/1145	ref	ref
Availability of resources for sanitation	No	30/176	0.70 [0.41 - 1.19]	0.76 [0.46 - 1.23]
	Yes	191/1076	ref	ref
Clinical capacity				
Capacity to perform cultures	No	44/247	0.82 [0.53 - 1.28]	0.96 [0.63 - 1.47]
	Yes	177/1005	ref	ref
Compliance with clinical & laboratory assessments				
Respiration rate[1]	Low	55/306	1.05 [0.68 - 1.63]	0.97 [0.64 - 1.46]
	High	166/946	ref	ref
White blood cell count	Low	59/317	0.92 [0.59 - 1.43]	1.06 [0.71 - 1.58]
	High	162/935	ref	ref
Mental status[1]	Low	13/111	0.65 [0.32 - 1.31]	0.67 [0.35 - 1.30]
	High	208/1141	ref	ref
Blood pressure[1]	Low	7/72	0.46 [0.18 - 1.20]	0.50 [0.21 - 1.20]
	High	214/1180	ref	ref
Individual characteristics				
Enrolment	From another hospital	60/162	3.94 [2.52 - 6.15]	3.78 [2.47 - 5.79]
	Already hospitalized	95/579	1.46 [1.00 - 2.12]	1.39 [0.98 - 1.98]
	From home	66/511	ref	ref

Maternal pre-existing condition	No	189/1169	ref	ref
	Yes	32/83	3.22 [1.93 - 5.38]	3.09 [1.89 - 5.04]
Previous births	> 2	60/245	2.37 [1.40 - 4.02]	2.20 [1.47 - 3.28]
	1 or 2	93/467	1.76 [1.19 - 2.61]	1.75 [1.24 - 2.48]
	0	68/540	ref	ref
Mode of end of pregnancy	Abortion	51/228	1.71 [1.09 - 2.69]	1.58 [1.03 - 2.42]
	Caesarean birth	107/617	1.30 [0.89 - 1.89]	1.14 [0.80 - 1.61]
	Vaginal birth	63/407	ref	ref
Anaemia	No	133/870	ref	ref
	Yes	88/382	1.50 [1.07 - 2.10]	1.77 [1.28 - 2.44]
Age in years (x)[2]	x+1	-	0.99 [0.97 - 1.02]	1.03 [1.01 - 1.05]

*Severe maternal outcome includes maternal near-miss and death

cOR: crude odds ratio
aOR: adjusted odds ratio
CI: confidence interval
LIC: low-income country; LMIC: lower-middle income country; UMHIC: upper-middle/high-income country
BEmONC: basic emergency obstetric and newborn care

[1] Measures used for the qSOFA score for identifying high-risk patients for sepsis-related mortality
[2] As compared to women one year younger
